# Supplementary material for: The Putative C2H2 Transcription Factor VadH Governs Development, Osmotic Stress Response, and Sterigmatocystin Production in Aspergillus nidulans
Source: Cells. 2022 Dec 10;11(24):3998. doi: 10.3390/cells11243998 (PMC9776899; doi:10.3390/cells11243998)
Supplement: Supplementary file 1 [file cells-11-03998-s001.zip › cells-2031641-supplementary.pdf]

Table S1. *Aspergillus* strains used in this study.

Strains	Relevant genotype	References
FGSC4	*A. nidulans* wild type, *veA*$^{+}$	FGSC[a]
RJMP1.59	*pyrG89*; *pyroA4*; *veA*$^{+}$	[21]
TNJ36	*pyrG89*; *AfupyG*$^{+}$; *pyroA4*; *veA*$^{+}$	[22]
Δ*vosA*	*pyrG89*; *pyroA4*; Δ*vosA*::*AfupyG*$^{+}$; *veA*$^{+}$	[13]
Δ*velB*	*pyrG89*; *pyroA4*; Δ*velB*::*AfupyG*$^{+}$; *veA*$^{+}$	[13]
Δ*vadH*	*pyrG89; pyroA4;* Δ*vadH::AfupyG+; veA+*	This study
C'*vadH*	*pyrG89; pyroA::vadH(p)::vadH::FLAG3x::pyroAb;* Δ*vadH::AfupyrG+; veA+*	This study
OE*vadH*	*pyrG89; AfupyrG+; pyroA::nii(p)::vadH::FLAG::pyroA; veA+*	This study

[a] Fungal Genetic Stock Center

Table S2. Primers used in this study.

Name	Sequence (5'-3') [a]	Purpose
OXL-1	AAACGGTGGATGCTCAGTGGTCG	5' flanking region of vadH
OXL-2	*GCTTTGGCCTGTATCATGACTTCA*GATGATAAAG GCGGACCCTGCGT	5' vadH with AfupyrG tail
OXL-3	*ATCGACCGAACCTAGGTAGGGTA*GTTTCCCAGTT CGTGCGTTGTCG	3' vadH with AfupyrG tail
OXL-4	TCATTGTTACTACTCGGCCTCGG	3' flanking region of vadH
OXL-5	CCGCATCGAGGTACAGCAAAGGT	5' nested of vadH
OXL-6	TACATCTCACGAAGCACACACTG	3' nested of vadH
OXL-7	GCCATGCCGTTGGTGCTGTTGCT	verification of vadH ORF
OXL-8	ACGCCCCTTGATGCCCTTGTTAC	verification of vadH ORF
OXL-9	CTGGTGTCTCCCCTGAACTTTGT	verification of 5' flanking region
OXL-10	TTGCTGTTGCCAGGTGAGGGTAT	verification of 5' flanking region
OXL-11	**GATAAGCAAAGTGGACTGATAGC**	verification of 3' flanking region
OXL-12	**AGTCCTTTAGGCAGCAGTGATAG**	verification of 3' flanking region
OXL-15	GG**GGTACC**CAGTCAAGCGACAACCTCTACTC	vadH complement *Kpn* I-pHS13
OXL-16	CCC**AAGCTT**CACTGAGTGACAAGCTCTCACAC	vadH complement *Hin*d III-pHS13
OXL-31	CG**GAATTC**ATGGAGACCGCAGAGCCTGTATC	vadH overexpression *Eco*R I –pHS11
OXL-32	CCC**AAGCTT**CTCGTCACCGCGCTTCTCAATGA	vadH overexpression *Hin*d III –pHS11
OHS-694	GCTGAAGTCATGATACAGGCCAAA	5' AfupyrG marker
OHS-695	ATCGTCGGGAGGTATTGTCGTCAC	3' AfupyrG marker
VadH-QF	CAACATCACAATCACCATCACC	qRT-PCR for VadH
VadH-QR	TCATGAGCAGATACTCCGTTAC	qRT-PCR for VadH
Actin-QF	GGCAACATCGTTATGTCTGG	qRT-PCR for actin
Actin-QR	CCGATCCAGACGGAGTATTT	qRT-PCR for actin
AN9219-QF	GTCGCAATGTGTCTTCGTAAAC	qRT-PCR for AN9219
AN9219-QR	CACCGAGAACTTCATTTTGTGT	qRT-PCR for AN9219
AN7030-QF	GTCATCAAGGCTCTCTACGATT	qRT-PCR for AN7030
AN7030-QR	CTGATAACGTGGAAGAAATCGC	qRT-PCR for AN7030
AN1443-QF	CTACAAGTCGGCTTCACCTATG	qRT-PCR for AN1443
AN1443-QR	GGAACATCCTGTGTCTTAGGAG	qRT-PCR for AN1443
AN1951-QF	CCATCCCACCTCCAAACTT	qRT-PCR for AN1951
AN1951-QR	TCATCGCTTATCCCCCAAAG	qRT-PCR for AN1951
AN4190-QF	CATCGAGACCATCTACCAGAC	qRT-PCR for AN4190
AN4190-QR	GATAGTGACGGTCGAGTAAGTG	qRT-PCR for AN4190
AN9473-QF	AATGGTTCCTACGAATTTGCTG	qRT-PCR for AN9473
AN9473-QR	ATAACACTGAATGTAAAGCGGC	qRT-PCR for AN9473
AN6273-QF	TCTTTCCTACGACACCAAGTAC	qRT-PCR for AN6273
AN6273-QR	GAGGTGTAAGTGATCTGGTAGC	qRT-PCR for AN6273
AN0554-QF	GTCACTTCCGACATGAAGATTG	qRT-PCR for AN0554
AN0554-QR	CCTGGTACGAGATCATGTTGTA	qRT-PCR for AN0554
AN1941-QF	AACAACATGGGTATTTTGGCTC	qRT-PCR for AN1941
AN1941-QR	GCCATGTTAGATTGTGTTCTGG	qRT-PCR for AN1941

AN10218-QF	TTGCCTACGGTATCAATATGCA	qRT-PCR for AN10218
AN10218-QR	CTCGTTGATTTGCCATCTGTAC	qRT-PCR for AN10218
AN8043-QF	GATACTGTTATACCGCACCTCA	qRT-PCR for AN8043
AN8043-QR	TAATCTTGTGCACATTGCTGAC	qRT-PCR for AN8043
AN10694-QF	GGGGAAATGCAGATTATTGTCG	qRT-PCR for AN10694
AN10694-QR	GCGAATTTTCAGACTCCCATTT	qRT-PCR for AN10694
AN0773-QF	TCACGCGAATAATCCTTGTTTC	qRT-PCR for AN0773
AN0773-QR	TTTCTTGATGTTTGCCCATAGC	qRT-PCR for AN0773
AN0493-QF	TGGACCATTACTTCCCTTAAGC	qRT-PCR for AN0493
AN0493-QR	TAGGTACAGTCGGTAGAGTCG	qRT-PCR for AN0493
AN3804-QF	TATCATTGACGGAAAGGTCGTT	qRT-PCR for AN3804
AN3804-QR	GAAGTAGATGTGGACTCAGTGG	qRT-PCR for AN3804
AN8438-QF	CATTTGCGAGAGACGTTTACAC	qRT-PCR for AN8438
AN8438-QR	CGAGCAAATAAGAAGCCGTATC	qRT-PCR for AN8438
AN1460-QF	GTTTTTGAGCAGAGTGGAGATC	qRT-PCR for AN1460
AN1460-QR	ACTGCACAACTTTTTGATCCAG	qRT-PCR for AN1460
AN10326-QF	GTCTTGATGGCGATGTTCTTAC	qRT-PCR for AN10326
AN10326-QR	GGACAATTCATTGGCGATATCC	qRT-PCR for AN10326
AN2924-QF	GTTCGAGGCTGCTACTACTATC	qRT-PCR for AN2924
AN2924-QR	GAATCATATAGGATTGCGGCAC	qRT-PCR for AN2924
AN10003-QF	CGTACAGACAGCAAATCTGAAG	qRT-PCR for AN10003
AN10003-QR	GATCGAAAATTGTCTCCCCATG	qRT-PCR for AN10003
AN2002-QF	CAGTGCTCTCTTTAGCTATGGT	qRT-PCR for AN2002
AN2002-QR	GATAATCACCATTGCGCAGTAG	qRT-PCR for AN2002
AN2928-QF	GTTTCCACGAACATCATTAGCG	qRT-PCR for AN2928
AN2928-QR	GAAAGAGAGAAGACCGTGAAGA	qRT-PCR for AN2928
AN11378-QF	GATCTGTTGTTCGGGATACTGT	qRT-PCR for AN11378
AN11378-QR	CAACGCACTTATTTCCATAGCA	qRT-PCR for AN11378
AN3804-QF	TATCATTGACGGAAAGGTCGTT	qRT-PCR for AN3804
AN3804-QR	GAAGTAGATGTGGACTCAGTGG	qRT-PCR for AN3804
AN9168-QF	TCAAGATGCCTACGGATTATAC	qRT-PCR for AN9168
AN9168-QR	GACCGACTCTCTCACCATAAA	qRT-PCR for AN9168
AN11039-QF	GAAAAAGATCCCGGTTACAAGG	qRT-PCR for AN11039
AN11039-QR	GTTGAACTGGAGGTCAGACTTA	qRT-PCR for AN11039
AN9443-QF	CAAGCTTTCAAGATCAAAACGC	qRT-PCR for AN9443
AN9443-QR	TATCCTGAGACTGTGCACAAAT	qRT-PCR for AN9443
AN10385-QF	CTTGATACGTCGTTATTGTCCG	qRT-PCR for AN10385
AN10385-QR	CTTGTGAAAATGACGACCGTAG	qRT-PCR for AN10385
AN7836-QF	AAGACGGATAATTTTGCACAGG	qRT-PCR for AN7836
AN7836-QR	TTGGTCCTCATTAGTCTTCCAG	qRT-PCR for AN7836
AN2242-QF	CTACTGACTGCAAATTCGTGAG	qRT-PCR for AN2242
AN2242-QR	AATGGAGCAGATCGTGGTAATG	qRT-PCR for AN2242

[a] Tail sequences are shown in italics. Restriction enzyme sites are in bold

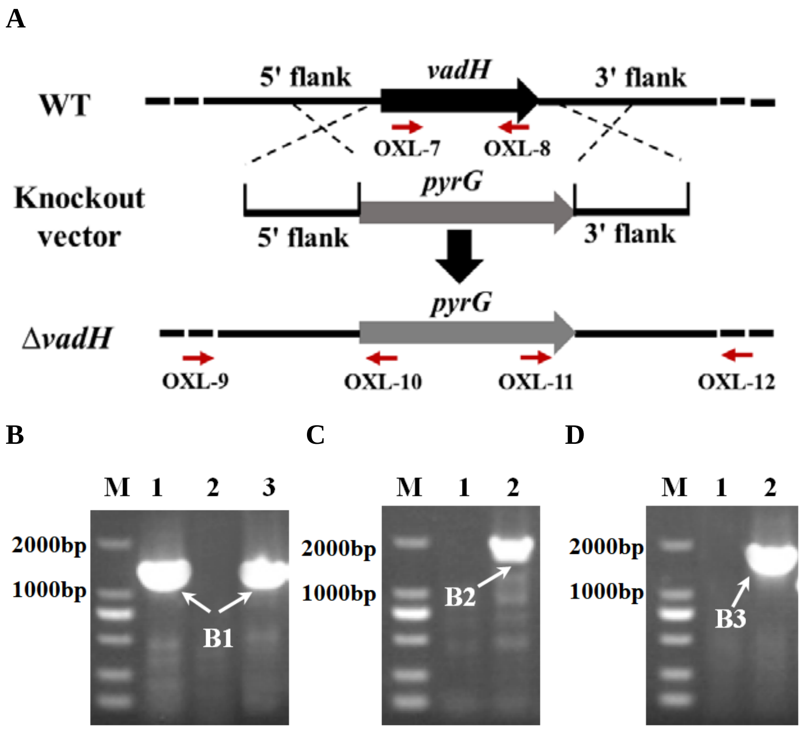

Figure S1. Verification of the gene-knockout mutant and complementary strain. (A) The verification strategy of *vadH*. (B) PCR verification results of OXL7 / OXL8. (C) PCR verification results of OXL9 / OXL10; (D) PCR verification results of OXL11 / OXL12. M: DL2000 DNA marker, 1: WT, 2: Δ*vadH*, 3: C' *vadH*. B1: PCR amplification fragment using the primers OXL7 / OXL8; B2: PCR amplification fragment using the primers OXL9 / OXL10; B3: PCR amplification fragment using the primers OXL11 / OXL12.

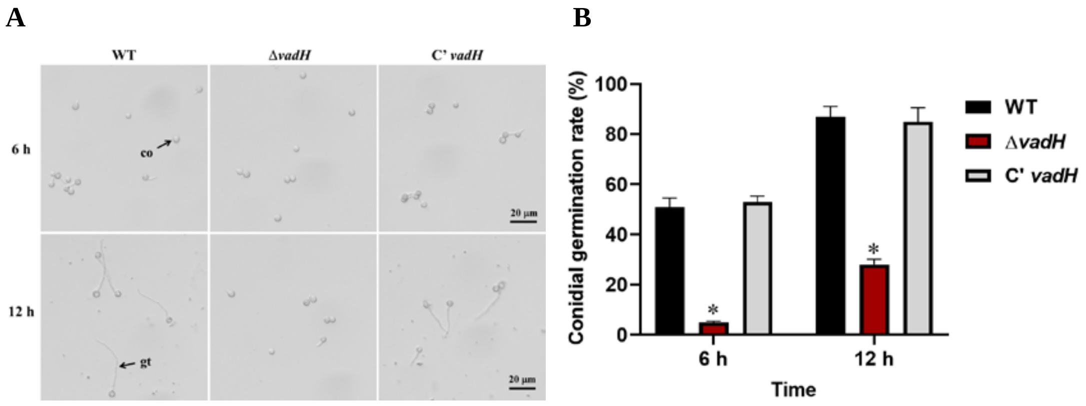

Figure S2. Conidial germination of WT, Δ*vadH* and the complemented strain. (A) Conidial germination at 6 and 12 h. co: conidium, gt: germ tube. (B) Statistical analyses of conidial germination rates. The asterisks represent significant level (* *p<0.05*).

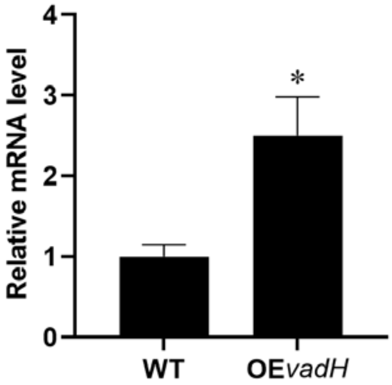

Figure S3. Verification of the mRNA level of *vadH* in OE*vadH*.

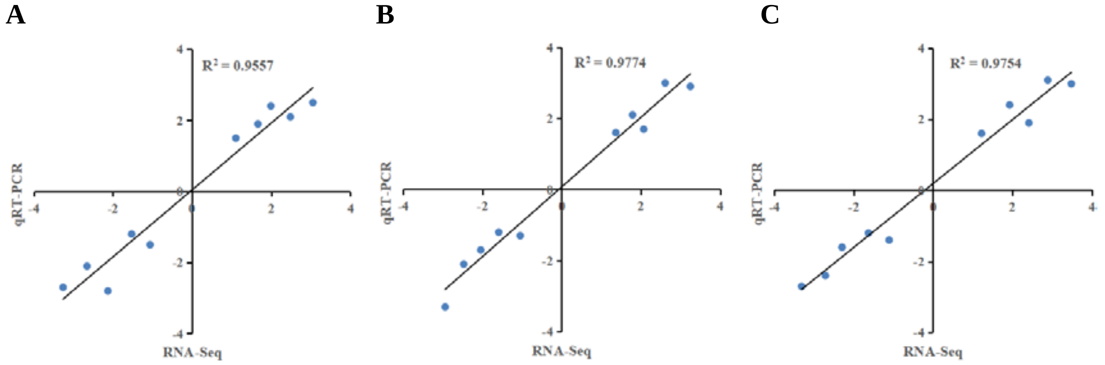

Figure S4. The qRT-PCR verification of RNA-Seq data in three stages. Five up-regulated and five downregulated genes were randomly selected and validated. (**A**) The qRT-PCR verification of RNA-Seq data in the vegetative growth stage. The selected DEGs for vegetative grow stage were as follows: AN9219 (log_2Fold change in RNA-Seq: 3.05), AN7030 (2.48), AN1443 (1.99), AN1951 (1.66), AN4190 (1.10), AN9473 (-1.06), AN6273 (-1.53), AN0554 (-2.13), AN1941 (-2.66) and AN10218 (-3.26). (**B**) The qRT-PCR verification of RNA-Seq data in the asexual development stage. The selected DEGs for asexual development stage were as follows: AN8043 (3.25), AN10694 (2.61), AN0773 (2.07), AN0493 (1.79), AN3804 (1.37), AN8438 (-1.04), AN1460 (-1.59), AN10326 (-2.04), AN2924 (-2.47) and AN10003 (-2.94). (**C**) The qRT-PCR verification of RNA-Seq data in the sexual development stage. The selected DEGs for sexual development stage were as follows: AN2002 (3.48), AN2928 (2.89), AN11378 (2.42), AN3804 (1.93), AN9168 (1.22), AN11039 (-1.11), AN9443- (-1.63), AN10385 (-2.29), AN7836 (-2.71) and AN2242 (-3.31).
